# Effect of high-dose intravenous vitamin C on inflammation in cancer patients

**DOI:** 10.1186/1479-5876-10-189

**Published:** 2012-09-11

**Authors:** Nina Mikirova, Joseph Casciari, Andrea Rogers, Paul Taylor

**Affiliations:** 1 Riordan Clinic, 3100 North Hillside, Wichita, KS, USA

## Abstract

**Background:**

An inflammatory component is present in the microenvironment of most neoplastic tissues. Inflammation and elevated C-reactive protein (CRP) are associated with poor prognosis and decreased survival in many types of cancer.

Vitamin C has been suggested as having both a preventative and therapeutic role in a number of pathologies when administered at much higher-than-recommended dietary allowance levels.

Since in vitro studies demonstrated inhibition of pro-inflammatory pathways by millimolar concentrations of vitamin C, we decided to analyze the effects of high dose IVC therapy in suppression of inflammation in cancer patients.

**Methods:**

45 patients with prostate cancer, breast cancer, bladder cancer, pancreatic cancer, lung cancer, thyroid cancer, skin cancer and B-cell lymphoma were treated at the Riordan Clinic by high doses of vitamin C (7.5 g -50 g) after standard treatments by conventional methods.

CRP and tumor markers were measured in serum or heparin-plasma as a routine analysis. In addition, serum samples were collected before and after the IVCs for the cytokine kit tests.

**Results:**

According to our data positive response to treatment, which was demonstrated by measurements of C- reactive protein, was found in 75% of patients and progression of the inflammation in 25% of patients. IVC treatments on all aggressive stage cancer patients showed the poor response of treatment.

There was correlation between tumor markers (PSA, CEA, CA27.29 and CA15-3) and changes in the levels of C-reactive protein.

Our test of the effect of IVC on pro-inflammatory cytokines demonstrated that inflammation cytokines IL-1α, IL-2, IL-8, TNF-α, chemokine eotaxin and CRP were reduced significantly after treatments.

**Conclusions:**

The high dose intravenous ascorbic acid therapy affects C-reactive protein levels and pro-inflammation cytokines in cancer patients. In our study, we found that modulation of inflammation by IVC correlated with decreases in tumor marker levels.

In summary, our data support the hypothesis that high dose intravenous ascorbate treatments may reduce inflammation in cancer patients. Our results suggest that further investigations into the use of IVC to reduce inflammation in diseases where inflammation is relevant are warranted.

## Background

Inflammation plays a key role in tumour development, affecting tumour proliferation, angiogenesis, metastasis, and resistance to therapy [1-6]. Key features of cancer-related inflammation (CRI) include leukocyte infiltration, cytokine build-up, tissue remodelling, and angiogenesis. Infiltrating leukocytes such as tumour associated macrophages (TAMs), neutrophils, dendritic cells, and lymphocytes establish an inflammatory microenvironment [7] and are key components in tumours of epithelial origins [8]. These leukocytes secrete pro-inflammatory cytokines such as IL1, IL6, TNFα, TGFβ, FGF, EGF and HGF21, as well as chemokines such as CCL2 and CXCL8 [9]. While immune cells may repress tumour growth in some cases [10-12], there is increasing concern that inflammatory microenvironments caused by infiltrating leukocytes can facilitate cancer development [13-16]. In clinical studies, TAMs are associated with poor prognosis, while the use of anti-inflammatory agents is associated with reduced instances of certain cancers [14,17].

Several studies indicate that inflammation is a marker of high cancer risk and poor treatment outcome [18-22]. In response to systemic inflammation, and in particular in response to elevated IL-6 levels, the liver produces CRP [23], a protein that binds to dead or dying cells to activate the complement system. CRP can be used as a marker of systemic inflammation. It correlates with disease progression and can be used to monitor infection [18,24-27]. For example, subjects with highly elevated CRP concentrations (above 80 mg/L, as compared to values below 5 mg/L) showed 3.5 times the risk for “all-cause” mortality compared to other subjects [28]. There are particularly strong negative correlations between CRP levels and cancer survival [14,24,28-38] in a wide variety of cancer types. For example, cancer patients with highly elevated CRP showed increased mortality by a factor of 28. Thus, CRP concentration data confirm a correlation between cancer progression and inflammation.

Serum CRP concentrations in human subjects are, according to one report, inversely correlated with antioxidant nutrient concentrations [39]. Vitamin C (ascorbate, ascorbic acid, AA) is a water-soluble antioxidant and essential nutrient for immune cells and extracellular matrix production [40,41]. These properties, as well as the correlations between ascorbate depletion in cancer patients and prognosis [42], suggest that vitamin C may have a beneficial effect on inflammation in cancer patients. Experiments by Hartel et al. [43] indicate that 20 mM ascorbate inhibited production of IL-6 and TNF-α in monocytes without affecting IL-1 or IL-8 levels. For lymphocytes, the same ascorbate concentrations inhibited IL-2 production without affecting TNF-α of IFN-γ levels. Ascorbate, at milli molar concentrations, may also inhibit NF-κB activation in endothelial cells [44]. NF-κB is an important transcription factor that mediates changes in gene expression during inflammation. The effect may be concentration dependent, as a different study indicated that low ascorbate doses (0.2 mM) actually enhanced NF-κB in Jurkat T-cells [45]. Other studies show that ascorbate inhibits TNF-α activation of NF-κB in human cell lines in vitro in a concentration dependent fashion, and can also inhibit GM-CSF, IL-3, and IL-5 production [46].

While millimolar ascorbate concentrations are not usually considered ‘physiological’, they can be achieved if the vitamin is administered intravenously at high dose. Intravenous vitamin C therapy has been used in the treatment of cancer [47,48]. Rationales for IVC therapy include preferential toxicity of ascorbate toward cancer cells [49,50], potential benefits of ascorbate for immune cells [48], and ascorbate inhibitory effect on angiogenesis [51,52]. In a study with guinea pigs, tumour growth was significantly reduced in cases where intra-tumour ascorbate concentrations reached the millimolar level [53].

In addition, inflammation and oxidative stress can cause down-regulation of immune system associated with T cell dysfunction, which has been described in cancer, infectious, and autoimmune diseases. In cancer and other inflammatory conditions, the T cell receptor (TCR) zeta chain is cleaved resulting in T-cell and Natural Killer (NK) cell inability to activate [54]. Several studies have shown that chronic inflammation is mandatory for the induction of an immunosuppressive environment, due to the high concentrations of reactive oxygen species formed by myeloid suppressor cells [55,56]. Clinically, this occurrence is associated with poor prognosis [57,58]. As the in vitro tissue cultures have shown a reversal of TCR cleavage by antioxidants [59,60], the result of treatment by ascorbic acid may augment T-cell and NK cell immunity.

Over the course of 35 years, the Riordan Clinic has administered IVC to cancer patients. For some of these subjects, blood chemistry data before, during, and after IVC treatments provide measurements of plasma ascorbate, CRP levels, and the levels of cancer markers such as PSA and CEA. This manuscript details an analysis of these clinical data to determine if a correlation exists between IVC treatments, inflammation (measured by CRP, cytokine levels), and tumour progression (measured by PSA, CEA and other tumour markers).

## Methods

### Biochemical assays and analysis

CRP concentrations in blood (serum or heparin-plasma) were analysed using a particle-enhanced immune-turbidimetric assay (CRP Ultra WR Reagent kit, Genzyme) according to manufacturer’s instructions on an automated analyser (CobasMIRA, Roche Diagnostics). The upper boundary for the normal range was set to 1.9 mg/L.

Blood concentrations of cytokines INF-γ, IL-1, IL-2, IL-8, TNF-α, and eotaxin were obtained using commercially available cytokine array ELISA kits (Ray Biotech). The chemiluminescent signal was imaged by the BioImage system (Alpha Innotech). The antigen-antibody spots on the image were circled by Spot Denso of FluorChem SP software (Alpha Innotech). Serum samples from non-cancer people were used as normal ranges. The pro-inflammatory markers from eleven patients before and after 6 IVCs were compared.

Serum vitamin C measurements were attained as described elsewhere [61].

### Statistical analysis

The data were analysed by Systat software (Systat, Inc) and Kaleidagraph software. Variables were presented as mean values ± SD, or as medians with corresponding 25th percentiles. Association between different factors was assessed using linear models. Statistical significance was accepted if the null hypothesis could be rejected at p ≤ 0.05.

### Patients and treatments

The diagnosis of patients as to their malignant disease and status was based on the information provided to Riordan Clinic and sent from pathologist and/or oncologist at the request of the patient.

First, patients are tested for glucose-6-phosphate dehydrogenase deficiency prior to treatment, as this deficiency can cause hemolysis. Patients with G6PDH deficiency were not given IVC. For eligible patients, the Riordan IVC protocol was administered as described elsewhere [48]. Briefly, subjects initially receive of 7.5 to 15 g ascorbate infused by slow drip in saline solution. To ensure that patient has adequate renal function, hydration and urinary voiding capacity, baseline lab tests were performed that include a serum chemistry profile and urinalysis. Provided these first treatments are well tolerated, patients are given the option to continue with 25 to 50 g infusions up to three times per week.

Patients who received IVC treatment signed informed consent. Privacy and patient anonymity guidelines were strictly followed in gathering the data for analysis.

Data were analysed for 45 patients who went through the Riordan IVC protocol: twenty-four with prostate cancer, nine with breast cancer, three each with bladder cancer, pancreatic cancer, and lung cancer, and one each with thyroid cancer, skin cancer and B-cell lymphoma. Several patients had metastasis. Treatment and additional clinical data were obtained from medical records. Many of the patients were treated initially by conventional way, by operation, radiotherapy, and administration of hormones and cytotoxic substances. Characteristics of subjects under analysis are listed in Table 1: the median age of the patients was 68 years, with a range of 47–85 years. Clinical response to IVC treatment was assessed by screening parameters of inflammation, cancer markers, complete blood counts, lipid profiles and nutritional status. The median follow-up time was 7.2 years and ranged from 1 year to 18 years.

**Table 1:** Characteristics of subjects under analysis with duration and number of treatments

**Type of cancer**	**Age**	**Sex**	**CRP before**	**CRP after**	**Days of treatment**	**Number of treatments**	**Conventional treatment**
Anorectal adenocarcinoma, grade 3, invasive	60	F	19.6	1.9	663	4	low anterior resection, colorectostomy
Biliary cancer, cholangiocarcinoma S-III(IV)	77	M	19.58	143.76	68	33	No records (NR)
Bladder - transitional cell carcinoma x 2, grade 3	66	M	57.5	11	76	7	operations, post treatment cycles
Bladder, grade 3	65	M	6.09	0.94	167	7	2 times surgery
Breast	71	M	6.2	3.1	179	2	mastectomy, Tomoxifen
Breast	57	F	19.4	0.4	512	47	NR
Breast	82	F	22	8.9	132	2	radiation, 36 treatments
Breast	66	F	12	2.25	193	3	Lamoxifin - 1.5 year
Breast	89	F	4.1	8.5	454	19	NR
Breast, ductal carcinoma in situ, high nuclear grade	67	F	21.1	5.5	555	12	no surgery
Breast carcinoma in situ, Basal cell carcinoma, Lymphoma	71	F	133.2	2.3	682		surgery, mastectomy, chemotherapy
Breast, category 5	78	F	8.6	13.1	379	20	NR
Breast, poorly differentiated duct cell carcinoma grade 4, Pancreas, malignant stage III, nonresectable	78	F	3.7	8.9	233	4	no radiation or chemotherapy, mastectomy
Breast, infiltrating tubular carcinoma	77	F	2.7	7.9	232	3	no radiation, chemotherapy or mastectomy
Breast, stage 1, T1 OMO, grade 2	53	F	12	3.8	44	11	lumpectomy
Breast, Stage IIA; Nottingham grade 2	69	F	9.3	3	121	20	breast lumpectomy; partial mastectomy; excision (L) breast;
Colon, stage IV, liver cancer	64	M	44	3.4	232	12	surgery, chemotherapy, hepatic resection
Gastric cancer	70	M	1.6	59.8	159	15	NR
Large B cell lymphoma	25	F	10.5	0.3	82	18	NR
Lung	65	M	216	35.9	174	27	NR
Lung - squamous cell, grade 2	80	M	9	6.7	82	20	surgery, no chemotherapy
Lung, renal	82	F	19.2	59	167	37	NR
Lymphoma	53	F	10.8	5.2	110	5	Immune therapy 4 treatments/week every 6 months, Rutuxin
Pancreas	85	M	3.8	54.5	42	11	NR
Pancreas, breast	71	F	10.1	0.4	119	44	NR
Pancreas, liver and bone metastasis	89	M	2	25	423		cholecystojejunostomy and gastrojejunostomy
Pros, Gleason score 6-8	82	M	3.9	8.8	279	8	no surgery or hormonal therapy
Prostate	77	M	26.2	0.4	261	16	NR
Prostate	80	M	153.3	148.4	11	5	NR
Prostate	93	M	29.2	2.1	136	20	NR
Prostate, colon cancer	89	M	43.4	31.5	113	5	chemotherapy, colonectomy
Prostate, Gleason score 2.5, malignant skin melanoma	72	M	6	0.9	140	8	NR
Prostate, Gleason score 6	53	M	8.4	1.9	38	4	surgery
Prostate, Gleason score 6, squamous cell melanoma, bone cancer	78	M	35.5	1.3	23	4	radical prosectomy
Prostate, Gleason score 6, stage T2A	72	M	22.1	3.66	400		surgery
Prostate, Gleason score 7	78	M	16.7	4.8	401	4	radiation and hormonal therapy
Prostate, pancreas	74	M	14.9	40.7	89	24	NR
Prostate, pancreas	86	M	500	54.3	542	102	NR
Prostate, stage I	65	M	2.53	8.59	82	1	prostatectomy
Prostate, Gleason score 4, increased to 6–9 during 2 years	81	M	5	0.6	1449	3	NR
Prostate, Esophageal cancer	84	M	15.3	3.1	37	6	removal of esophagus
Prostate, metastatic adenocarcinoma, Gleason score 4 + 4 = 8	74	M	25	0.7	791	7	radical prostatectomy
Rectal adenocarcinoma, invasive through muscle layer	71	M	2.2	7.2	137	2	chemotherapy radiation
Renal, Basal cell carcinoma	82	M	30.5	19.5	48	10	operation, 33 treatments by radiation
Skin, B-cell Lymphoma, Lung	83	M	8.9	1.5	754	36	surgery, 40 radiations

To analyse the effect of IVC on the level of pro-inflammatory cytokines, IVC was given to 11 new coming cancer patients at dose of 15 g, 25 g, 50 g, 50 g, 50 g, and 50 g each time for 6 times. All 11 patients were at relative clinical stable status after the conventional cancer treatments (surgery, chemo, or radiation therapies). Patients had different types of cancers (breast, colon, lung, pancreatic, renal and prostate cancer). For example, all breast-cancer patients in the ascorbate-treated group already had mastectomy and radiotherapy and had been given hormones, but all had relapsed by the time ascorbate supplementation was commenced. Treatment of this group of patients was assigned by IVC 15 g-50 g once a day one time per week. Sera were collected before IVC, before the 6th IVC and after the 6th IVC for the cytokine kit test to see the trend of IVC effects on cytokines. Six patients were tested further for the effect of 50 g IVC infusion on C-reactive protein.

## Results

**Figure 1:**
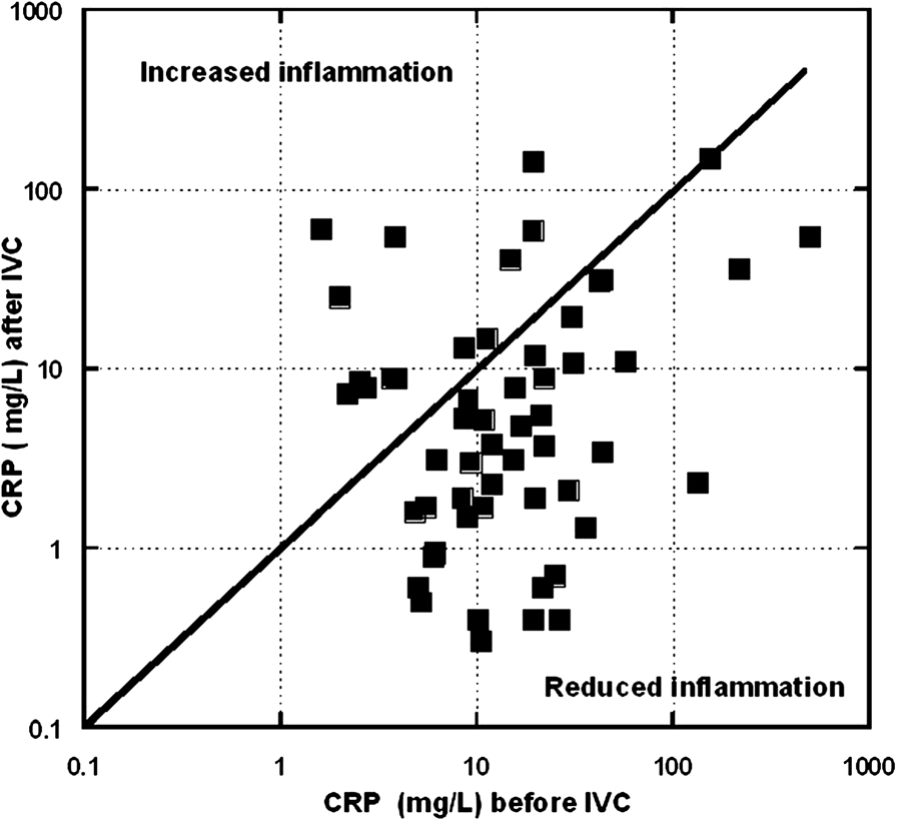
**C-reactive protein levels after intravenous vitamin C treatments are compared to CRP levels before treatments. **Data points (squares) under the diagonal line demonstrate a positive effect of treatment introducing CRP levels. Data points above the diagonal indicate CRP increases after treatment.

Our database contained forty-five subjects who received IVC therapy (ranging from one to 100 treatments, with a median of nine treatments and an inter-quartile range, IQR, of five to eighteen treatments) and for whom CRP data were available before or after treatment (Table 1). Figure 1 shows CRP values before and after treatment.

Most of the subjects, 76 ± 13% (95% confidence) showed a reduction in CRP values during IVC therapy. If only subjects who had initially elevated CRP (> 10 mg/L) levels are considered, 86 ± 13% (95% confidence) showed a reduction in CRP values during IVC therapy. This is also indicated by the fact that twenty-eight subjects had elevated CRP prior to therapy, but only 14 had elevated CRP levels after treatment. Among the twenty-eight subjects who started with elevated CRP levels, the median CPR reduction was 80% with an IQR of 39% to 94%.

**Figure 2:**
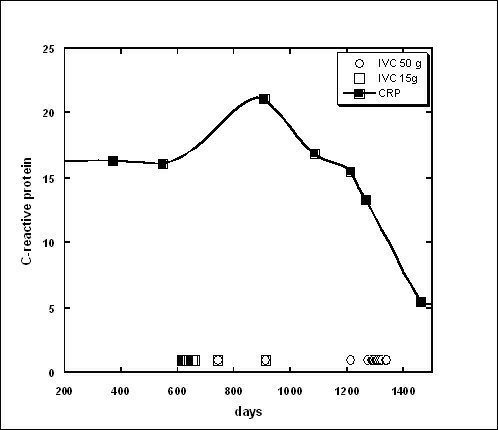
**Tracking of CRP concentration (solid squares and curve) over time in a 67 year old subject with ductal carcinoma. **The subject was given ten IVC treatments of 15 g (time of treatment indicated by open squares near the x axis) and eleven treatments of 50 g (indicated by open circles near the x axis). Treatments were typically given weekly. CRP concentration decreased from an initial value of 16 mg/L level to a final value of 5.5 mg/L after the last treatment.

Example of the changed levels of CRP with indicated periods of IVC treatments by 15 g and 50 g of IVC is shown in Figure 2.

Unfortunately, our data covers patients who varied in the number of treatments they received. For patients with prostate cancer, the percentage decrease in CRP concentration after treatment was higher when the patient had more frequent treatments. Median CRP reductions of 95% were attained in subjects who were treated frequently (intervals between IVC infusions being fewer than 10 days) while those treated less frequently. The negative response to IVC treatment with dosages 15 g-50 g was found in patients with gastric cancer (CRP was increased from 1.0 mg/L to 60 mg/L in 160 days, 15 IVC treatments). Progression was measured for three patients with pancreatic cancer. One patient had metastasis in liver and bones, and for this patient CRP was increased from 3.8 mg/L to 54 mg/L in 42 days (13 IVC treatments by dosages 25 g-50 g). For another patient without metastasis, the increase in CRP was from 2 mg/L to 25 mg/L in 400 days. The third patient with pancreatic cancer had 24 treatments during 90 days and CRP was increased from 15 mg/L to 89 mg/L. The patient with cholangiocarcinoma stage IV had 33 IVC treatments in 68 days and CRP was increased from 19 mg/L to 144 mg/L. Negative outcome was found for a patient with lung cancer with metastasis. This patient had 37 IVCs during 170 days and CRP was increased from 19 mg/L to 59 mg/L.

Progression was found for 2 patients with breast cancer (one with infiltrating tubular carcinoma, poorly differentiated duct cell carcinoma with second diagnosis pancreatic cancer in malignant stage 3, non-resectable), and another with breast cancer, category 5. For these patients the increase was mild from 3 mg/L and 8 mg/L to 10 mg/L and 13 mg/L frequently showed median CRP reductions of 64%.

**Figure 3:**
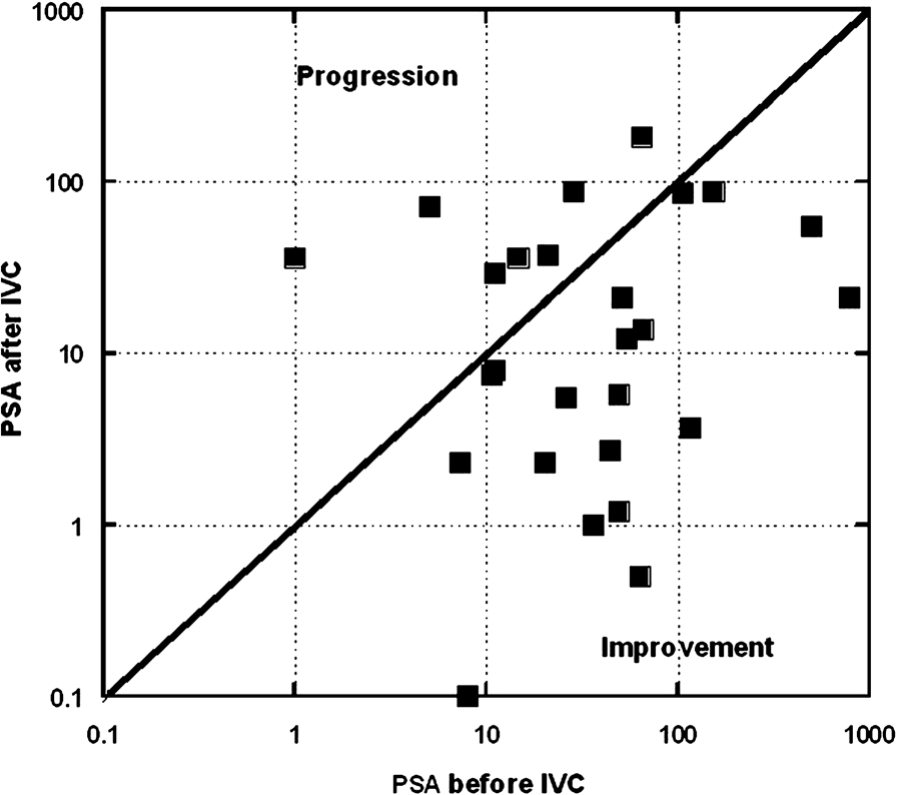
**Changes in PSA levels after IVC treatments are compared to PSA levels before treatments. **Data points (squares) under the diagonal line demonstrate a positive effect of treatment in reducing PSA levels. Data points above the diagonal line indicate PSA increases after treatment.

**Figure 4:**
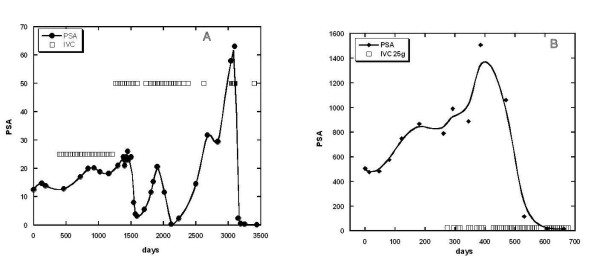
**(A, B) – Tracking of PSA levels (circles, squares and curve) over time in two subjects with prostate cancer. **Subjects were given IVC treatments at times indicated by the open squares. Subject A: initial Gleason score = 4; treatments typically given monthly at doses of either 25 g (34 treatments) or 50 g (38 treatments); At maximum PSA values (~ 60 ng/mL) treatments were given weekly. PSA levels decreased from initial values of 10–60 ng/mL to final values of 1–2.4 ng/mL. Subject B: initial Gleason score = 6–9; treatments typically given weekly at doses of either 7.5 g (2 treatments) or 25 g (40 treatments); PSA levels decreased from maximum values of 1500 ng/mL to a final value of 7 ng/mL.

**Figure 5:**
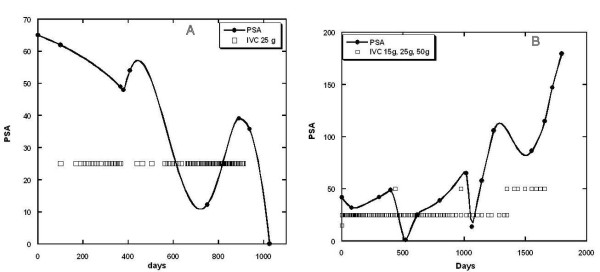
**(A, B) - Tracking of PSA levels (solid circles and curve) over time in two subjects with prostate cancer. **Subjects were given IVC treatments at times indicated by the open squares. Subject A: initial Gleason score =6; treatments typically given weekly or twice weekly at doses of 25 g; PSA levels decreased from initial values of 60 ng/ml to final values in the normal range. Subject B: initial Gleason score = 6 – 8; treatments typically given weekly or twice weekly at doses of either 25 g (140 treatments) or 50 g (7 treatments); PSA levels rose and fell during treatments, and then experienced a steep increase after treatment was discontinued.

The effects of IVC therapy on PSA tumor marker levels is shown in Figure 3. We had twenty patients for whom PSA levels were assessed before and after IVC therapy. Fifteen of these subjects, 75% with a 95% confidence interval of ± 19%, showed reduced PSA levels during IVC treatment. The examples of the positive response to IVC treatments for patients with prostate cancer are presented in Figures 4 and 5.

We also had data from nineteen other subjects (mostly breast cancer patients, with a few lung, pancreatic, bladder, and colon cancer patients) for cancer markers CEA, CA27.29, or CA15.3. There was statistically no benefit here, with only 53 ± 22% (95% confidence) of subjects showing reduction in these tumor markers post treatments.

Table 2 summarizes the percentage of patients who saw improvement in various parameters. The fraction of subjects seeing reduced inflammation, as measured by decreases in CRP, was significantly above fifty percent in the prostate cancer group, the breast cancer group, and the entire study group. IVC seemed to have a beneficial effect in reducing PSA levels, but we could not see a significant effect in breast cancer subjects or other cancer markers.

**Table 2:** Percentage of patients who saw reduction in CRP or tumour marker levels after IVC therapy “ ± ” indicates 95% confidence intervals, “†” indicates proportions significantly above 50%

**Cancer Type**	**N**	**% Improvement**	
**CRP Reduction**			
Prostate	14	79 ± 21	†
Breast	10	80 ± 25	†
All patients	45	76 ± 13	†
**Tumour Marker Reduction**			
Prostate	18	77 ± 21	†
All PSA	20	75 ± 19	†
Breast	11	73 ± 26	
All CA/CEA	19	53 ± 22	
All patients	40	65 ± 15	†

**Figure 6:**
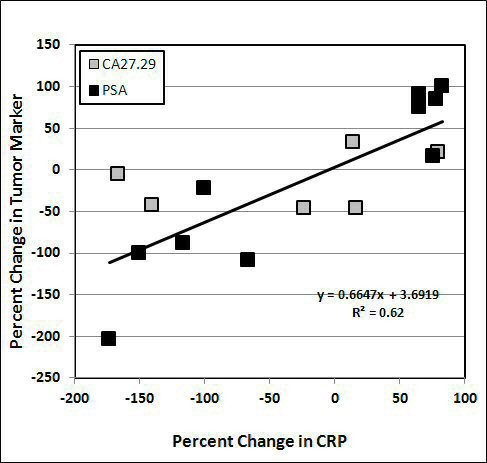
**Correlation between changes in CRP levels and changes in tumour markers after IVC therapy. **Linear regression indicates a positive correlation (R^2^ = 0.62) between changes in CRP and changes in the values of tumour markers PSA and CA 27.29. Data excluded several cases of aggressive tumours when the changes in CRP and tumour markers were higher than 300%.

In patients for whom both tumor marker and CRP data are available, we found an interesting correlation: patients who had reduced CRP levels tended to also have reduced tumor marker values. This is shown in Figure 6: linear regression shows a positive correlation with r^2^ = 0.62. Note that we did not observe a correlation between CRP concentrations and tumor marker values pre-treatments (r^2^ = 0.02)

**Figure 7:**
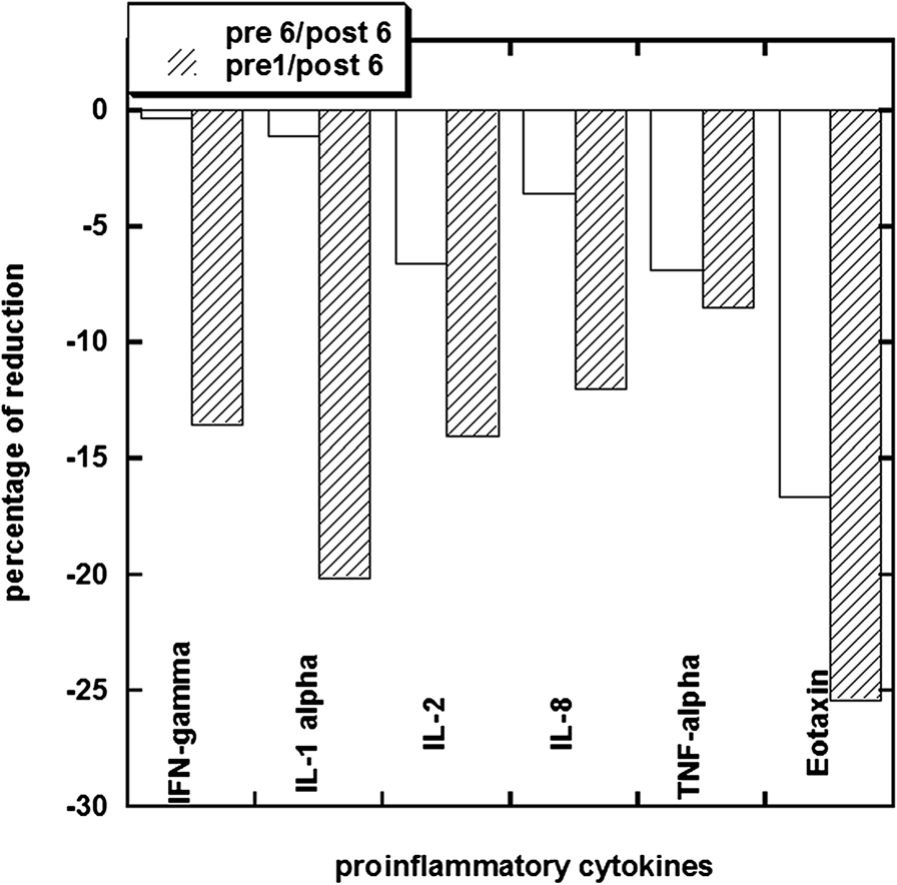
**Percentage of reduction in serum cytokine levels after IVC therapy. **Data present the changes in the level of proinflamatory cytokines after treatment by single dosage of 50 g IVC (pre6/post6) and after 6 treatments by IVC with dosages 15 g-50 g (pre1/post6). In all cancer patients initial level of vitamin C in plasma was low (0.9-1.2 mg/dl) and increased to the levels 200–300 mg/dL after last IVC. In all cases, proinflammatory cytokine levels decreased during IVC treatment.

To study the effects of IVC on inflammation in more detail, we measured serum cytokine levels in eleven subjects before and after IVC therapy. These subjects were given six treatments (at doses of 15 g for the first treatment, 25 g for the second treatment, and 50 g for the next four treatments). Plasma ascorbate levels in these subjects were measured to verify that sufficient concentrations were attained for cytokine effects. At the fifty gram treatments, plasma ascorbate concentrations immediately post infusion reached average values of roughly 18 mM. The effects of these treatments on serum concentrations of key pro-inflammatory cytokines are shown in Figure 7. This figure shows two endpoints for each cytokine. The first is the change in cytokine concentration at the point just before the patients are given their sixth IVC dose and after this single 50 g IVC. The second is the change in cytokine concentration (relative to pre-therapy value) at the point just after the patients are given all six IVC dosages. The first endpoint perhaps indicates sustained reductions in cytokine levels due to one treatment while the second indicates an effect of ascorbate after several dosing. After six treatments, patients had noticeably lower levels of IL-2, TNF-α, and eotaxin. All cytokine levels were reduced after the last IVC injection. For six of these subjects, CRP levels were measured before and after the last injection of IVC. In all six cases, CRP concentrations decreased, typically by ten percent, after the infusion.

## Discussion

The vitamin C is used in complementary oncology, with intravenous administration being of particular interest since it allows for plasma ascorbate concentrations an order of magnitude beyond those attainable with oral administration [62,63]. Clinical studies suggest that this approach is feasible and potentially beneficial [47,48,64,65]. Several mechanisms of action for ascorbate efficacy against cancer have been proposed over the years, but to our knowledge its potential role in mediating inflammation has not been previously addressed. In the research described in this manuscript, we used CRP as a clinical inflammation marker. Twenty-eight out of forty-five subjects in our study had sharply elevated CRP levels (above 10 mg/L) prior to IVC therapy, suggesting that inflammation is a prevalent problem for cancer patients. This is especially important since other reports indicate that inflammation, particularly elevated CRP, is a marker of a poor prognosis [66]. In 76 ± 13% of our subjects, IVC therapy reduced CRP levels, with improvements being more prevalent (86 ± 13%) in the sub-population with pre-treatment CRP above 10 mg/L. Post-treatment, the number of subjects with these sharply elevated CRP levels was reduced from 28 to 14.

We found that decreases in CRP during treatment correlated with tumour marker decreases. This is consistent with other work [67] demonstrating that plasma CRP levels are well-correlated with serum PSA levels in prostate cancer patients. In the study [67] it was hypothesized that prostatic infection and inflammation may increase serum prostate-specific antigen levels. In addition, authors suggest that plasma CRP measurements may help to differentiate benign conditions from prostate cancer in patients with elevated serum PSA levels. Inflammation might be fundamental in prostate cancer and chronic inflammation may be a legitimate target for prostate cancer chemoprevention and treatment. Inflammatory processes also play a role in the development of histologic benign prostatic hyperplasia [68,69]. Acute and chronic inflammatory infiltrates are routinely found in prostate tissue specimens obtained from men with BPH, and greater levels of inflammation have been observed in larger prostates [70-73].

It is interesting in our study, therefore, that prostate cancer patients showed the most benefit (in terms of reduced PSA and CRP levels) from IVC therapy. A potential effect of IVC in reducing inflammation is also supported by our serum cytokine data, which demonstrate that levels of pro-inflammatory cytokines decrease during IVC therapy. IL-2, TNF-α, and eotaxin appeared to be chronically reduced in patients getting IVC therapy, while all six cytokines studied (IL-1α, IFN-γ, and IL-8, in addition to IL-2, TNF-α, and eotaxin) were acutely reduced after ascorbate infusions of 50 g. Average depression of IL-1 was 20% for six patients and the average decrease for eotaxin was 25%. Interleukin-1 is known to promote inflammatory processes and augment metastasis [74]. It is abundant at tumour sites, where it affects the process of carcinogenesis, tumour growth and invasiveness, and the patterns of tumour-host interactions [75,76]. IL-1 induces uPA expression and NF-kB activation. TNF-alpha, another key inflammatory cytokines, plays a central role in the tumour progression. Constitutive expression of the TNF-alpha from tumour microenvironment is a characteristic of many malignant tumours and its presence is often associated with poor prognosis [77-80]. Eotaxin-1 (CLL11) is a chemo-attractant and lymphocyte activator that has been shown to affect tumour cell growth [81-83]. Chemokine receptor expression in many cancers correlates with poor prognosis, and there is evidence that eotaxin induces angiogenesis and metastasis [84].

In summary, our analysis of data from cancer patients given IVC, along with our tests of cytokine levels, suggest that IVC may reduce inflammation in cancer patients, and that this reduction in inflammation is correlated with reductions in the tumour markers PSA.

The strength of the study is that we used the protocol established in our clinic for the treatment of cancer patients by IVC therapy, lacking at many clinics at least as adjuvant therapy in treatment of malignant disease, and demonstrated the effect of this treatment on the level of inflammation in cancer patients. We analysed the array of patients with various forms of malignant disease, although there were more patients with prostate malignancies than other forms of malignant disease.

The limitations of the study are that the measurements of parameters of inflammation and tumour markers were not very detailed and many patients did not have measurements of the inflammation cytokines during treatment.

Further research in this area and clinical studies of the efficacy of intravenous high dose vitamin C are warranted.

## Competing interests

The authors declare that they have no competing interests.

## Authors’ contributions

NAM, PRT, AR and JJC analyzed data, interpreted results of analysis and conceptualized the manuscript. All authors read and approved the final manuscript.
